# Bis{4-phenyl-1-[1-(pyridin-2-yl-κ*N*)ethyl­idene]thio­semicarbazidato-κ^2^ *N* ^1^,*S*}cadmium

**DOI:** 10.1107/S1600536812016558

**Published:** 2012-04-21

**Authors:** Alexandra de Souza Fonseca, Vanessa Carratu Gervini, Leandro Bresolin, Aline Locatelli, Adriano Bof de Oliveira

**Affiliations:** a Escola de Química e Alimentos, Universidade Federal do Rio Grande, Av. Itália km 08, Campus Carreiros, 96203-900 Rio Grande-RS, Brazil; b Departamento de Química, Universidade Federal de Santa Maria, Av. Roraima, Campus, 97105-900 Santa Maria-RS, Brazil; c Departamento de Química, Universidade Federal de Sergipe, Av. Marechal Rondon s/n, Campus, 49100-000 São Cristóvão-SE, Brazil

## Abstract

The reaction of cadmium acetate dihydrate with 2-acetyl­pyridine (4-phenyl­thio­semicarbazone) yielded the title compound, [Cd(C_14_H_13_N_4_S)_2_]. The Cd^II^ atom is six-coordin­ated in a distorted octa­hedral environment by two deproton­ated thio­semicarbazone ligands acting in a tridentate chelating mode through two N and one S atoms, forming metalla-rings. In the crystal, mol­ecules are connected through inversion centers *via* pairs of N—H⋯S inter­actions, building a one-dimensional hydrogen-bonded polymer along [0-1-1].

## Related literature

For the synthesis of 2-acetyl­pyridine-(4-phenyl­thio­semi­carbazone), see: Offiong & Martelli (1997 ▶). For thio­semi­carbazone complexes with similar coordination environments, see: Ali *et al.* (2002 ▶); Kovala-Demertzi *et al.* (2005 ▶). For the anti­bacterial and anti­fungal activity of Cd^II^ thio­semicarbazone complexes, see: Alomar *et al.* (2010 ▶). For the bioinorganic chemistry of the Cd^II^ ion and its relation to biologically important ions, namely Zn^II^ and Ca^II^, see: Kaim & Schwederski (1995 ▶).
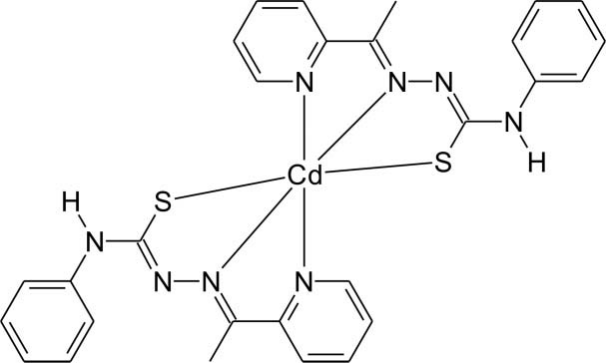


## Experimental

### 

#### Crystal data

[Cd(C_14_H_13_N_4_S)_2_]
*M* *_r_* = 651.09
Triclinic, 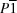

*a* = 9.8452 (2) Å
*b* = 13.0116 (3) Å
*c* = 13.1736 (5) Å
α = 116.495 (1)°
β = 105.757 (1)°
γ = 96.122 (1)°
*V* = 1401.81 (7) Å^3^
*Z* = 2
Mo *K*α radiation
μ = 0.96 mm^−1^
*T* = 296 K
0.22 × 0.14 × 0.11 mm

#### Data collection

Bruker APEXII CCD diffractometer
Absorption correction: multi-scan (*SADABS*; Bruker, 2009 ▶) *T*_min_ = 0.816, *T*_max_ = 0.902
29421 measured reflections
8199 independent reflections
5975 reflections with *I* > 2σ(*I*)
*R*_int_ = 0.044

#### Refinement

*R*[*F* ^2^ > 2σ(*F* ^2^)] = 0.038
*wR*(*F* ^2^) = 0.080
*S* = 1.06
8199 reflections
362 parameters
H atoms treated by a mixture of independent and constrained refinement
Δρ_max_ = 0.75 e Å^−3^
Δρ_min_ = −0.49 e Å^−3^

### 

Data collection: *APEX2* (Bruker, 2009 ▶); cell refinement: *SAINT* (Bruker, 2009 ▶); data reduction: *SAINT*; program(s) used to solve structure: *SHELXS97* (Sheldrick, 2008 ▶); program(s) used to refine structure: *SHELXL97* (Sheldrick, 2008 ▶); molecular graphics: *DIAMOND* (Brandenburg, 2006 ▶); software used to prepare material for publication: *publCIF* (Westrip, 2010 ▶).

## Supplementary Material

Crystal structure: contains datablock(s) I, global. DOI: 10.1107/S1600536812016558/bt5877sup1.cif

Structure factors: contains datablock(s) I. DOI: 10.1107/S1600536812016558/bt5877Isup2.hkl

Additional supplementary materials:  crystallographic information; 3D view; checkCIF report

## Figures and Tables

**Table 1:** Hydrogen-bond geometry (Å, °)

*D*—H⋯*A*	*D*—H	H⋯*A*	*D*⋯*A*	*D*—H⋯*A*
N4—H8⋯S1^i^	0.84 (2)	2.60 (3)	3.437 (2)	172 (2)
N8—H21⋯S2^ii^	0.83 (3)	2.79 (3)	3.513 (2)	147 (2)

Symmetry codes: (i) 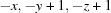; (ii) 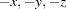.

**Fig. 1.**
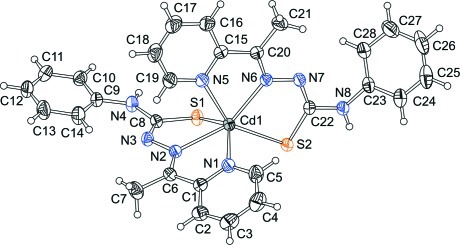
: The molecular structure of the title compound with labeling and displacement ellipsoids drawn at the 40% probability level.

**Fig. 2.**
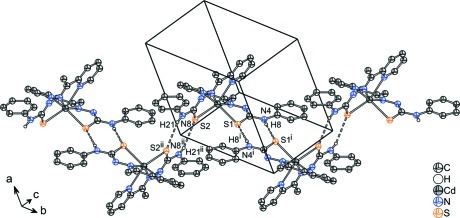
: The crystal structure of the title compound showing the molecules connected through N—H···S interactions and building a one-dimensional H-bonded polymer along the (0 11) direction. Hydrogen bonding is indicated as dashed lines and the figure is simplified for clarity. Symmetry codes: (i) -x, -y + 1, -z + 1; (ii) -x, -y, -z.

## supplementary crystallographic information

### Comment

Thiosemicarbazone derivatives have a wide range of applications on biological inorganic chemistry. For example, some Cd^II^ thiosemicarbazone complexes show important antibacterial and antifungal activity against *Acinetobacter baumannii* and *Aspergillus fumigatus*, respectively (Alomar *et al.*, 2010). In addition, the Cd^II^ ion has similarity with two biologically important elements, namely Zn^II^ and Ca^II^. The ionic radii for these cations are 0,95 Å, 0,74 Å and 1,00 Å, respectively, and the chemical similarity suggests that Cd^II^ may displace Zn^II^ and Ca^II^ from critical biological sites (Kaim & Schwederski, 1995), making the cadmium coordination chemistry very attractive. As part of our study of thiosemicarbazone derivatives, we report herein the synthesis and the crystal structure of a new Cd^II^ complex with 2-acetylpyridine-(4-phenylthiosemicarbazone).

In the title compound, in which the molecular structure unit matches the asymmetric unit, the Cd^II^ ion is six-coordinated in a distorted octahedral environment by two deprotonated 2-acetylpyridine-(4-phenylthiosemicarbazone) ligands acting in a tridentate chelating mode, forming five-membered rings (Fig. 1). The selected bond angles formed between donor atoms trough the Cd1 atom are N6—Cd1—N2 = 151.43 (7)°, N1—Cd1—S1 = 134.61 (5)° and N5—Cd1—S2 = 143.06 (5)° and show clearly a distorted octahedral environment. The displacement from ideal coordination geometry is probably a consequence of the geometrical requirements of the ligand and of crystal packing interactions.

The acidic hydrogen of the hydrazine fragment is lost by the reaction with KOH, which is in agreement for thiosemicarbazone derivatives prepared from aldehydes or ketones. The negative charge is delocalized over the C—N—N—C—S fragment as indicated by their intermediate bond distances. The imine and thioamide C—N distances indicate considerable double bond character, while the C—S distance is consistent with increased single bond character. For the first ligand, these distances are C6—N2 = 1.285 (3) Å, N2—N3 = 1.385 (2) Å, N3—C8 = 1.311 (3) Å and C8—S1 = 1.748 (2) Å and for the second ligand, they are C20—N6 = 1.291 (3) Å, N6—N7 = 1.373 (3) Å, N7—C22 = 1.305 (3) Å and C22—S2 = 1.738 (2) Å.

The two ligands are coordinated to the Cd^II^ ion in their meridional conformations with the S1/S2 thiolate and the N1/N5 pyridyl atoms *cis* to each other, while the N2/N6 azomethine atoms are *trans* to each other. A similar meridional conformation has also been observed in the Mn^II^ complex with the same thiosemicarbazone ligand (Kovala-Demertzi *et al.*, 2005) and in several Cd^II^ complexes with tridentate "NNS"-chelating ligands derived from thiosemicarbazones (Ali *et al.*, 2002). The two ligands show a Z—E—*Z* conformation for the donor atoms about the C1—C6/C6—N2/N3—C8 and the C15—C20/C20—N6/N7—C22 bonds, respectively.

The ligands are not planar (Fig. 1 and Fig. 2), the mean deviations from the least squares planes for the chelated fragments Cd1/N1/C1/C6/N2 and Cd1/N2/N3/C8/S1 amount to -0,0644 (16) ° for C1 and -0,2702 (12) ° for N2, respectively, and the dihedral angle between the two planes is 20,15 (10)°. The maximal deviation from the least squares plane through all non-hydrogen atoms for the acetylpyridine derivative fragment C1/C2/C3/C4/C5/C6/C7/N1 and for the phenyl-thiosemicarbazone derivative fragment C8/C9/C10/C11/C12/C13/C14/N2/N3/N4/S1 amount to 0,1120 (19) Å for C7 and 0,1301 (16) Å for N2, respectively, and the dihedral angle between the two planes is 30,76 (08)°.

Additionally, the mean deviations from the least squares planes for the chelated fragments Cd1/N5/C15/C20/N6 and Cd1/N6/N7/C22/S2 amount to 0,0489 (13) Å for N5 and 0,0371 (12) Å for N6, respectively, and the dihedral angle between the two planes is 4,31 (07)°. The maximal deviation from the least squares plane through all non-hydrogen atoms for the acetylpyridine derivative fragment C15/C16/C17/C18/C19/C20/C21/N5 and for the phenyl-thiosemicarbazone derivative fragment C22/C23/C24/C25/C26/C27/C28/N6/N7/N8/S2 amount to 0,0457 (17) Å for C21 and -0,5623 (31) Å for C28, respectively, and the dihedral angle between the two planes is 14,77 (11)°. The displacement from planarity, for both of the ligands, is probably a consequence of the coordination with the Cd^II^ ion and of crystal packing interactions.

The crystal packing is stabilized by intermolecular hydrogen bonding. The molecules are connected through inversion centers *via* pairs of N—H···S interactions (Table 1; N4—H8···S1^i^ and N8—H21···S2^ii^), building a one-dimensional H-bonded polymer along the (0 11) direction (Fig. 2). Symmetry codes: (i) -*x*, -*y* + 1, -*z* + 1; (ii) -*x*, -*y*, -*z*.

### Experimental

Starting materials were commercially available and were used without further purification. The synthesis of 2-acetylpyridine-(4-phenylthiosemicarbazone) was adapted from a procedure reported previously (Offiong & Martelli, 1997). 2-Acetylpyridine-(4-phenylthiosemicarbazone) (2 mmol) was dissolved in ethanol and treated with one KOH pellet. Stirring was maintained for 30 min, while the reaction mixture turns yellow. A solution of cadmium acetate dihydrate (1 mmol) also in ethanol was added under continuous stirring and under slight warming to 60 °C. After 3 h a yellow solid was formed. This solid was filtered-off, washed with small portions of cool ethanol and dried at room conditions. Yellow crystals of the complex, suitable for X-ray analysis, were obtained by recrystallization from a 2:1 mixture of acetone and dimethylformamide.

### Refinement

H atoms attached to C atoms were positioned with idealized geometry and were refined isotropic with *U*_eq_(H) set to 1.2 times of the *U*_eq_(C) for the aromatic and 1.5 times of the *U*_eq_(C) for methyl H atoms using a riding model with C—H = 0.93 Å and C—H = 0.96 Å, respectively. H atoms attached to N atoms were located in difference Fourier maps. Their positional and isotropic displacement parameters were refined.

### Crystal data

**Table:**

[Cd(C_14_H_13_N_4_S)_2_]	*Z* = 2
*M**_r_* = 651.09	*F*(000) = 660
Triclinic, *P*1	*D*_x_ = 1.543 Mg m^−^^3^
Hall symbol: -P 1	Mo *K*α radiation, λ = 0.71073 Å
*a* = 9.8452 (2) Å	Cell parameters from 5467 reflections
*b* = 13.0116 (3) Å	θ = 2.3–23.9°
*c* = 13.1736 (5) Å	µ = 0.96 mm^−^^1^
α = 116.495 (1)°	*T* = 296 K
β = 105.757 (1)°	Block, yellow
γ = 96.122 (1)°	0.22 × 0.14 × 0.11 mm
*V* = 1401.81 (7) Å^3^	

### Data collection

**Table:**

Bruker APEXII CCD diffractometer	8199 independent reflections
Radiation source: fine-focus sealed tube	5975 reflections with *I* > 2σ(*I*)
Graphite monochromator	*R*_int_ = 0.044
φ and ω scans	θ_max_ = 30.1°, θ_min_ = 2.7°
Absorption correction: multi-scan (*SADABS*; Bruker, 2009)	*h* = −13→13
*T*_min_ = 0.816, *T*_max_ = 0.902	*k* = −18→17
29421 measured reflections	*l* = −16→18

### Refinement

**Table:**

Refinement on *F*^2^	Primary atom site location: structure-invariant direct methods
Least-squares matrix: full	Secondary atom site location: difference Fourier map
*R*[*F*^2^ > 2σ(*F*^2^)] = 0.038	Hydrogen site location: inferred from neighbouring sites
*wR*(*F*^2^) = 0.080	H atoms treated by a mixture of independent and constrained refinement
*S* = 1.06	*w* = 1/[σ^2^(*F*_o_^2^) + (0.0314*P*)^2^ + 0.0548*P*] where *P* = (*F*_o_^2^ + 2*F*_c_^2^)/3
8199 reflections	(Δ/σ)_max_ = 0.001
362 parameters	Δρ_max_ = 0.75 e Å^−^^3^
0 restraints	Δρ_min_ = −0.49 e Å^−^^3^

### Special details

**Table:**

Geometry. All e.s.d.'s (except the e.s.d. in the dihedral angle between two l.s. planes) are estimated using the full covariance matrix. The cell e.s.d.'s are taken into account individually in the estimation of e.s.d.'s in distances, angles and torsion angles; correlations between e.s.d.'s in cell parameters are only used when they are defined by crystal symmetry. An approximate (isotropic) treatment of cell e.s.d.'s is used for estimating e.s.d.'s involving l.s. planes.
Refinement. Refinement of *F*^2^ against ALL reflections. The weighted *R*-factor *wR* and goodness of fit *S* are based on *F*^2^, conventional *R*-factors *R* are based on *F*, with *F* set to zero for negative *F*^2^. The threshold expression of *F*^2^ > σ(*F*^2^) is used only for calculating *R*-factors(gt) *etc*. and is not relevant to the choice of reflections for refinement. *R*-factors based on *F*^2^ are statistically about twice as large as those based on *F*, and *R*- factors based on ALL data will be even larger.

### Fractional atomic coordinates and isotropic or equivalent isotropic displacement parameters (Å^2^)

**Table:**

	*x*	*y*	*z*	*U*_iso_*/*U*_eq_	
Cd1	0.226151 (19)	0.208486 (15)	0.431225 (15)	0.03261 (6)	
S1	0.04751 (7)	0.34127 (6)	0.45863 (5)	0.03845 (15)	
S2	0.11139 (7)	0.07311 (6)	0.19832 (6)	0.04311 (16)	
C1	0.2081 (3)	0.0380 (2)	0.5482 (2)	0.0349 (5)	
C5	0.3030 (3)	−0.0431 (2)	0.3973 (3)	0.0542 (7)	
H4	0.3389	−0.0363	0.3415	0.065*	
C2	0.1985 (3)	−0.0638 (2)	0.5583 (3)	0.0468 (7)	
H1	0.1624	−0.0695	0.6145	0.056*	
C3	0.2432 (4)	−0.1567 (3)	0.4838 (3)	0.0582 (8)	
H2	0.2360	−0.2261	0.4886	0.070*	
C4	0.2977 (4)	−0.1462 (3)	0.4035 (3)	0.0622 (9)	
H3	0.3306	−0.2071	0.3541	0.075*	
C8	0.0828 (3)	0.3844 (2)	0.6114 (2)	0.0319 (5)	
C6	0.1612 (3)	0.1410 (2)	0.6249 (2)	0.0334 (5)	
C9	0.0826 (3)	0.5625 (2)	0.8007 (2)	0.0356 (5)	
C10	0.0613 (3)	0.6755 (2)	0.8373 (2)	0.0436 (6)	
H9	0.0400	0.7026	0.7818	0.052*	
C14	0.1168 (4)	0.5245 (3)	0.8844 (2)	0.0536 (8)	
H13	0.1325	0.4495	0.8618	0.064*	
C7	0.1277 (4)	0.1484 (3)	0.7318 (3)	0.0550 (8)	
H5	0.2132	0.1489	0.7887	0.083*	
H6	0.0495	0.0807	0.7050	0.083*	
H7	0.0989	0.2201	0.7704	0.083*	
C12	0.1050 (3)	0.7096 (3)	1.0382 (2)	0.0526 (7)	
H11	0.1124	0.7584	1.1179	0.063*	
C11	0.0713 (3)	0.7478 (2)	0.9550 (2)	0.0521 (7)	
H10	0.0551	0.8229	0.9782	0.063*	
C13	0.1275 (4)	0.5989 (3)	1.0025 (3)	0.0610 (8)	
H12	0.1505	0.5730	1.0589	0.073*	
N2	0.1521 (2)	0.22010 (16)	0.59191 (17)	0.0324 (4)	
N4	0.0634 (3)	0.49398 (19)	0.67666 (19)	0.0402 (5)	
N3	0.1191 (2)	0.32216 (17)	0.66504 (17)	0.0348 (4)	
N1	0.2593 (2)	0.04738 (17)	0.46709 (19)	0.0399 (5)	
C22	0.2505 (3)	0.1299 (2)	0.1648 (2)	0.0337 (5)	
C20	0.5236 (3)	0.3367 (2)	0.4314 (2)	0.0334 (5)	
C15	0.5532 (3)	0.3897 (2)	0.5636 (2)	0.0340 (5)	
C23	0.3274 (3)	0.0925 (2)	−0.0112 (2)	0.0405 (6)	
C24	0.3295 (3)	−0.0091 (3)	−0.1097 (2)	0.0503 (7)	
H22	0.2653	−0.0818	−0.1376	0.060*	
C16	0.6781 (3)	0.4789 (2)	0.6492 (2)	0.0489 (7)	
H14	0.7463	0.5080	0.6250	0.059*	
C28	0.4211 (3)	0.1993 (3)	0.0278 (3)	0.0581 (8)	
H26	0.4193	0.2683	0.0927	0.070*	
C19	0.4774 (3)	0.3928 (2)	0.7153 (2)	0.0489 (7)	
H17	0.4083	0.3634	0.7385	0.059*	
C18	0.5999 (3)	0.4811 (3)	0.8043 (3)	0.0600 (8)	
H16	0.6133	0.5106	0.8860	0.072*	
C17	0.7008 (3)	0.5246 (3)	0.7713 (3)	0.0642 (9)	
H15	0.7845	0.5845	0.8300	0.077*	
C25	0.4256 (4)	−0.0030 (4)	−0.1660 (3)	0.0688 (10)	
H23	0.4262	−0.0715	−0.2320	0.083*	
C27	0.5184 (4)	0.2041 (4)	−0.0295 (3)	0.0795 (11)	
H25	0.5827	0.2764	−0.0026	0.095*	
C26	0.5200 (4)	0.1022 (4)	−0.1263 (3)	0.0835 (12)	
H24	0.5858	0.1054	−0.1644	0.100*	
N6	0.3994 (2)	0.25909 (17)	0.35954 (17)	0.0327 (4)	
N5	0.4528 (2)	0.34715 (18)	0.59691 (18)	0.0389 (5)	
N7	0.3738 (2)	0.21135 (18)	0.23768 (18)	0.0374 (5)	
N8	0.2265 (3)	0.0800 (2)	0.0431 (2)	0.0429 (5)	
C21	0.6341 (3)	0.3730 (2)	0.3876 (3)	0.0480 (7)	
H18	0.6029	0.3237	0.3010	0.072*	
H19	0.7266	0.3639	0.4253	0.072*	
H20	0.6440	0.4548	0.4081	0.072*	
H8	0.041 (3)	0.531 (2)	0.639 (2)	0.035 (7)*	
H21	0.152 (3)	0.024 (2)	−0.002 (2)	0.042 (8)*	

### Atomic displacement parameters (Å^2^)

**Table:**

	*U*^11^	*U*^22^	*U*^33^	*U*^12^	*U*^13^	*U*^23^
Cd1	0.03545 (10)	0.03298 (10)	0.03154 (10)	0.00908 (7)	0.01550 (7)	0.01576 (8)
S1	0.0509 (4)	0.0407 (3)	0.0317 (3)	0.0229 (3)	0.0181 (3)	0.0201 (3)
S2	0.0417 (4)	0.0439 (4)	0.0312 (3)	−0.0027 (3)	0.0145 (3)	0.0108 (3)
C1	0.0361 (13)	0.0341 (13)	0.0347 (13)	0.0098 (10)	0.0081 (11)	0.0196 (11)
C5	0.071 (2)	0.0504 (17)	0.0535 (18)	0.0301 (15)	0.0326 (16)	0.0266 (15)
C2	0.0517 (17)	0.0423 (15)	0.0526 (17)	0.0155 (13)	0.0159 (14)	0.0295 (14)
C3	0.070 (2)	0.0413 (16)	0.067 (2)	0.0229 (15)	0.0158 (17)	0.0325 (16)
C4	0.076 (2)	0.0465 (17)	0.065 (2)	0.0355 (17)	0.0270 (18)	0.0236 (16)
C8	0.0342 (13)	0.0356 (12)	0.0323 (12)	0.0132 (10)	0.0161 (10)	0.0190 (11)
C6	0.0325 (13)	0.0377 (13)	0.0355 (13)	0.0118 (10)	0.0128 (10)	0.0217 (11)
C9	0.0387 (14)	0.0384 (13)	0.0318 (13)	0.0139 (11)	0.0160 (11)	0.0164 (11)
C10	0.0548 (17)	0.0375 (14)	0.0387 (15)	0.0139 (12)	0.0167 (13)	0.0188 (12)
C14	0.083 (2)	0.0494 (17)	0.0436 (16)	0.0323 (16)	0.0317 (16)	0.0268 (14)
C7	0.077 (2)	0.0605 (18)	0.0552 (18)	0.0314 (16)	0.0366 (17)	0.0407 (16)
C12	0.0600 (19)	0.0539 (18)	0.0330 (15)	0.0100 (15)	0.0200 (14)	0.0120 (14)
C11	0.0620 (19)	0.0373 (15)	0.0437 (16)	0.0114 (13)	0.0199 (14)	0.0092 (13)
C13	0.089 (3)	0.066 (2)	0.0376 (16)	0.0265 (18)	0.0281 (16)	0.0293 (16)
N2	0.0367 (11)	0.0310 (10)	0.0342 (11)	0.0130 (9)	0.0156 (9)	0.0176 (9)
N4	0.0582 (15)	0.0395 (12)	0.0352 (12)	0.0258 (11)	0.0220 (11)	0.0227 (11)
N3	0.0424 (12)	0.0341 (11)	0.0337 (11)	0.0164 (9)	0.0181 (9)	0.0177 (9)
N1	0.0485 (13)	0.0358 (11)	0.0429 (12)	0.0161 (10)	0.0226 (10)	0.0210 (10)
C22	0.0372 (13)	0.0355 (13)	0.0295 (12)	0.0119 (11)	0.0142 (10)	0.0151 (11)
C20	0.0298 (12)	0.0322 (12)	0.0374 (13)	0.0096 (10)	0.0113 (10)	0.0167 (11)
C15	0.0286 (12)	0.0319 (12)	0.0383 (14)	0.0100 (10)	0.0085 (10)	0.0164 (11)
C23	0.0376 (14)	0.0546 (16)	0.0310 (13)	0.0150 (12)	0.0143 (11)	0.0208 (12)
C24	0.0570 (18)	0.0598 (18)	0.0364 (15)	0.0242 (15)	0.0189 (13)	0.0225 (14)
C16	0.0342 (14)	0.0499 (16)	0.0455 (16)	0.0017 (12)	0.0088 (12)	0.0151 (14)
C28	0.066 (2)	0.0600 (19)	0.0418 (16)	0.0029 (16)	0.0255 (15)	0.0190 (15)
C19	0.0475 (17)	0.0543 (17)	0.0359 (15)	0.0064 (13)	0.0106 (13)	0.0193 (13)
C18	0.0511 (18)	0.070 (2)	0.0342 (15)	0.0070 (16)	0.0053 (14)	0.0136 (15)
C17	0.0414 (17)	0.068 (2)	0.0418 (17)	−0.0061 (15)	−0.0007 (14)	0.0074 (16)
C25	0.062 (2)	0.103 (3)	0.0435 (18)	0.043 (2)	0.0279 (17)	0.0286 (19)
C27	0.067 (2)	0.099 (3)	0.058 (2)	−0.012 (2)	0.0248 (18)	0.034 (2)
C26	0.062 (2)	0.142 (4)	0.050 (2)	0.024 (3)	0.0331 (19)	0.043 (2)
N6	0.0313 (11)	0.0344 (10)	0.0318 (11)	0.0082 (9)	0.0121 (9)	0.0156 (9)
N5	0.0353 (11)	0.0407 (12)	0.0343 (12)	0.0056 (9)	0.0088 (9)	0.0164 (10)
N7	0.0334 (11)	0.0444 (12)	0.0315 (11)	0.0061 (9)	0.0134 (9)	0.0164 (10)
N8	0.0394 (13)	0.0470 (13)	0.0313 (12)	0.0009 (11)	0.0133 (10)	0.0124 (11)
C21	0.0370 (15)	0.0528 (17)	0.0535 (17)	0.0046 (12)	0.0173 (13)	0.0268 (14)

### Geometric parameters (Å, º)

**Table:**

Cd1—N6	2.3288 (19)	C11—H10	0.9300
Cd1—N2	2.3691 (18)	C13—H12	0.9300
Cd1—N1	2.381 (2)	N2—N3	1.385 (2)
Cd1—N5	2.446 (2)	N4—H8	0.84 (2)
Cd1—S1	2.5778 (6)	C22—N7	1.305 (3)
Cd1—S2	2.5814 (7)	C22—N8	1.371 (3)
S1—C8	1.748 (2)	C20—N6	1.291 (3)
S2—C22	1.738 (2)	C20—C15	1.484 (3)
C1—N1	1.344 (3)	C20—C21	1.489 (3)
C1—C2	1.385 (3)	C15—N5	1.346 (3)
C1—C6	1.487 (3)	C15—C16	1.380 (3)
C5—N1	1.332 (3)	C23—C28	1.371 (4)
C5—C4	1.376 (4)	C23—C24	1.386 (3)
C5—H4	0.9300	C23—N8	1.407 (3)
C2—C3	1.381 (4)	C24—C25	1.367 (4)
C2—H1	0.9300	C24—H22	0.9300
C3—C4	1.360 (4)	C16—C17	1.383 (4)
C3—H2	0.9300	C16—H14	0.9300
C4—H3	0.9300	C28—C27	1.383 (4)
C8—N3	1.311 (3)	C28—H26	0.9300
C8—N4	1.365 (3)	C19—N5	1.335 (3)
C6—N2	1.285 (3)	C19—C18	1.373 (4)
C6—C7	1.498 (3)	C19—H17	0.9300
C9—C14	1.377 (4)	C18—C17	1.353 (4)
C9—C10	1.387 (3)	C18—H16	0.9300
C9—N4	1.413 (3)	C17—H15	0.9300
C10—C11	1.376 (3)	C25—C26	1.355 (5)
C10—H9	0.9300	C25—H23	0.9300
C14—C13	1.383 (4)	C27—C26	1.375 (5)
C14—H13	0.9300	C27—H25	0.9300
C7—H5	0.9600	C26—H24	0.9300
C7—H6	0.9600	N6—N7	1.373 (3)
C7—H7	0.9600	N8—H21	0.83 (3)
C12—C13	1.365 (4)	C21—H18	0.9600
C12—C11	1.373 (4)	C21—H19	0.9600
C12—H11	0.9300	C21—H20	0.9600
			
N6—Cd1—N2	151.43 (7)	N3—N2—Cd1	120.07 (13)
N6—Cd1—N1	111.91 (7)	C8—N4—C9	132.0 (2)
N2—Cd1—N1	67.80 (6)	C8—N4—H8	116.0 (16)
N6—Cd1—N5	68.06 (7)	C9—N4—H8	111.9 (16)
N2—Cd1—N5	83.38 (7)	C8—N3—N2	112.35 (18)
N1—Cd1—N5	94.00 (7)	C5—N1—C1	118.4 (2)
N6—Cd1—S1	113.46 (5)	C5—N1—Cd1	122.97 (18)
N2—Cd1—S1	71.90 (5)	C1—N1—Cd1	117.63 (15)
N1—Cd1—S1	134.61 (5)	N7—C22—N8	115.7 (2)
N5—Cd1—S1	101.36 (5)	N7—C22—S2	129.77 (18)
N6—Cd1—S2	75.54 (5)	N8—C22—S2	114.48 (18)
N2—Cd1—S2	132.65 (5)	N6—C20—C15	116.4 (2)
N1—Cd1—S2	93.71 (5)	N6—C20—C21	123.3 (2)
N5—Cd1—S2	143.06 (5)	C15—C20—C21	120.3 (2)
S1—Cd1—S2	98.58 (2)	N5—C15—C16	121.1 (2)
C8—S1—Cd1	95.23 (8)	N5—C15—C20	117.2 (2)
C22—S2—Cd1	96.70 (8)	C16—C15—C20	121.7 (2)
N1—C1—C2	121.2 (2)	C28—C23—C24	119.2 (3)
N1—C1—C6	116.9 (2)	C28—C23—N8	123.3 (2)
C2—C1—C6	121.9 (2)	C24—C23—N8	117.5 (2)
N1—C5—C4	123.3 (3)	C25—C24—C23	120.3 (3)
N1—C5—H4	118.3	C25—C24—H22	119.9
C4—C5—H4	118.3	C23—C24—H22	119.9
C3—C2—C1	119.0 (3)	C15—C16—C17	119.3 (3)
C3—C2—H1	120.5	C15—C16—H14	120.3
C1—C2—H1	120.5	C17—C16—H14	120.3
C4—C3—C2	119.8 (3)	C23—C28—C27	119.9 (3)
C4—C3—H2	120.1	C23—C28—H26	120.1
C2—C3—H2	120.1	C27—C28—H26	120.1
C3—C4—C5	118.2 (3)	N5—C19—C18	123.1 (3)
C3—C4—H3	120.9	N5—C19—H17	118.5
C5—C4—H3	120.9	C18—C19—H17	118.5
N3—C8—N4	119.1 (2)	C17—C18—C19	118.8 (3)
N3—C8—S1	127.34 (17)	C17—C18—H16	120.6
N4—C8—S1	113.48 (17)	C19—C18—H16	120.6
N2—C6—C1	115.1 (2)	C18—C17—C16	119.3 (3)
N2—C6—C7	124.1 (2)	C18—C17—H15	120.3
C1—C6—C7	120.7 (2)	C16—C17—H15	120.3
C14—C9—C10	119.0 (2)	C26—C25—C24	120.6 (3)
C14—C9—N4	125.1 (2)	C26—C25—H23	119.7
C10—C9—N4	115.9 (2)	C24—C25—H23	119.7
C11—C10—C9	120.7 (3)	C26—C27—C28	120.0 (3)
C11—C10—H9	119.7	C26—C27—H25	120.0
C9—C10—H9	119.7	C28—C27—H25	120.0
C9—C14—C13	119.5 (3)	C25—C26—C27	120.0 (3)
C9—C14—H13	120.3	C25—C26—H24	120.0
C13—C14—H13	120.3	C27—C26—H24	120.0
C6—C7—H5	109.5	C20—N6—N7	115.53 (19)
C6—C7—H6	109.5	C20—N6—Cd1	122.35 (16)
H5—C7—H6	109.5	N7—N6—Cd1	122.07 (14)
C6—C7—H7	109.5	C19—N5—C15	118.3 (2)
H5—C7—H7	109.5	C19—N5—Cd1	125.71 (18)
H6—C7—H7	109.5	C15—N5—Cd1	115.53 (15)
C13—C12—C11	119.2 (3)	C22—N7—N6	115.70 (19)
C13—C12—H11	120.4	C22—N8—C23	127.6 (2)
C11—C12—H11	120.4	C22—N8—H21	114.8 (18)
C12—C11—C10	120.2 (3)	C23—N8—H21	115.6 (18)
C12—C11—H10	119.9	C20—C21—H18	109.5
C10—C11—H10	119.9	C20—C21—H19	109.5
C12—C13—C14	121.5 (3)	H18—C21—H19	109.5
C12—C13—H12	119.3	C20—C21—H20	109.5
C14—C13—H12	119.3	H18—C21—H20	109.5
C6—N2—N3	117.64 (19)	H19—C21—H20	109.5
C6—N2—Cd1	121.55 (15)		
			
N6—Cd1—S1—C8	−124.64 (10)	N6—Cd1—N1—C1	155.39 (17)
N2—Cd1—S1—C8	25.28 (9)	N2—Cd1—N1—C1	6.27 (17)
N1—Cd1—S1—C8	53.53 (11)	N5—Cd1—N1—C1	87.45 (18)
N5—Cd1—S1—C8	−53.81 (10)	S1—Cd1—N1—C1	−22.8 (2)
S2—Cd1—S1—C8	157.47 (8)	S2—Cd1—N1—C1	−128.71 (17)
N6—Cd1—S2—C22	−2.87 (9)	Cd1—S2—C22—N7	2.4 (2)
N2—Cd1—S2—C22	−177.49 (10)	Cd1—S2—C22—N8	−178.32 (17)
N1—Cd1—S2—C22	−114.55 (10)	N6—C20—C15—N5	4.1 (3)
N5—Cd1—S2—C22	−12.87 (12)	C21—C20—C15—N5	−175.7 (2)
S1—Cd1—S2—C22	109.27 (8)	N6—C20—C15—C16	−176.0 (2)
N1—C1—C2—C3	0.3 (4)	C21—C20—C15—C16	4.3 (4)
C6—C1—C2—C3	179.7 (2)	C28—C23—C24—C25	1.0 (4)
C1—C2—C3—C4	1.0 (4)	N8—C23—C24—C25	−179.6 (3)
C2—C3—C4—C5	−1.6 (5)	N5—C15—C16—C17	0.4 (4)
N1—C5—C4—C3	1.0 (5)	C20—C15—C16—C17	−179.6 (2)
Cd1—S1—C8—N3	−31.2 (2)	C24—C23—C28—C27	−1.3 (5)
Cd1—S1—C8—N4	151.43 (17)	N8—C23—C28—C27	179.3 (3)
N1—C1—C6—N2	11.0 (3)	N5—C19—C18—C17	0.2 (5)
C2—C1—C6—N2	−168.4 (2)	C19—C18—C17—C16	−0.1 (5)
N1—C1—C6—C7	−169.2 (2)	C15—C16—C17—C18	−0.1 (5)
C2—C1—C6—C7	11.4 (4)	C23—C24—C25—C26	0.1 (5)
C14—C9—C10—C11	−1.3 (4)	C23—C28—C27—C26	0.7 (5)
N4—C9—C10—C11	177.3 (3)	C24—C25—C26—C27	−0.8 (6)
C10—C9—C14—C13	0.7 (4)	C28—C27—C26—C25	0.4 (6)
N4—C9—C14—C13	−177.7 (3)	C15—C20—N6—N7	179.82 (18)
C13—C12—C11—C10	−0.4 (5)	C21—C20—N6—N7	−0.4 (3)
C9—C10—C11—C12	1.1 (4)	C15—C20—N6—Cd1	2.1 (3)
C11—C12—C13—C14	−0.1 (5)	C21—C20—N6—Cd1	−178.17 (17)
C9—C14—C13—C12	0.0 (5)	N2—Cd1—N6—C20	−6.2 (3)
C1—C6—N2—N3	−175.47 (19)	N1—Cd1—N6—C20	−89.70 (19)
C7—C6—N2—N3	4.7 (4)	N5—Cd1—N6—C20	−4.35 (17)
C1—C6—N2—Cd1	−5.3 (3)	S1—Cd1—N6—C20	88.90 (18)
C7—C6—N2—Cd1	174.9 (2)	S2—Cd1—N6—C20	−177.89 (19)
N6—Cd1—N2—C6	−95.5 (2)	N2—Cd1—N6—N7	176.23 (14)
N1—Cd1—N2—C6	−0.13 (18)	N1—Cd1—N6—N7	92.71 (17)
N5—Cd1—N2—C6	−97.20 (19)	N5—Cd1—N6—N7	178.06 (18)
S1—Cd1—N2—C6	158.52 (19)	S1—Cd1—N6—N7	−88.69 (16)
S2—Cd1—N2—C6	73.6 (2)	S2—Cd1—N6—N7	4.52 (15)
N6—Cd1—N2—N3	74.4 (2)	C18—C19—N5—C15	0.1 (4)
N1—Cd1—N2—N3	169.78 (18)	C18—C19—N5—Cd1	−171.9 (2)
N5—Cd1—N2—N3	72.71 (16)	C16—C15—N5—C19	−0.4 (4)
S1—Cd1—N2—N3	−31.57 (15)	C20—C15—N5—C19	179.6 (2)
S2—Cd1—N2—N3	−116.53 (15)	C16—C15—N5—Cd1	172.36 (19)
N3—C8—N4—C9	4.0 (4)	C20—C15—N5—Cd1	−7.6 (3)
S1—C8—N4—C9	−178.4 (2)	N6—Cd1—N5—C19	178.4 (2)
C14—C9—N4—C8	−5.7 (5)	N2—Cd1—N5—C19	−2.5 (2)
C10—C9—N4—C8	175.8 (3)	N1—Cd1—N5—C19	−69.6 (2)
N4—C8—N3—N2	−172.7 (2)	S1—Cd1—N5—C19	67.5 (2)
S1—C8—N3—N2	10.1 (3)	S2—Cd1—N5—C19	−171.19 (17)
C6—N2—N3—C8	−166.8 (2)	N6—Cd1—N5—C15	6.18 (15)
Cd1—N2—N3—C8	23.0 (2)	N2—Cd1—N5—C15	−174.70 (17)
C4—C5—N1—C1	0.4 (4)	N1—Cd1—N5—C15	118.22 (17)
C4—C5—N1—Cd1	−168.1 (2)	S1—Cd1—N5—C15	−104.72 (16)
C2—C1—N1—C5	−1.0 (4)	S2—Cd1—N5—C15	16.6 (2)
C6—C1—N1—C5	179.6 (2)	N8—C22—N7—N6	−178.4 (2)
C2—C1—N1—Cd1	168.07 (19)	S2—C22—N7—N6	0.8 (3)
C6—C1—N1—Cd1	−11.3 (3)	C20—N6—N7—C22	177.8 (2)
N6—Cd1—N1—C5	−36.1 (2)	Cd1—N6—N7—C22	−4.5 (3)
N2—Cd1—N1—C5	174.8 (2)	N7—C22—N8—C23	10.8 (4)
N5—Cd1—N1—C5	−104.0 (2)	S2—C22—N8—C23	−168.6 (2)
S1—Cd1—N1—C5	145.7 (2)	C28—C23—N8—C22	−44.0 (4)
S2—Cd1—N1—C5	39.8 (2)	C24—C23—N8—C22	136.6 (3)

### Hydrogen-bond geometry (Å, º)

**Table:**

*D*—H···*A*	*D*—H	H···*A*	*D*···*A*	*D*—H···*A*
N4—H8···S1^i^	0.84 (2)	2.60 (3)	3.437 (2)	172 (2)
N8—H21···S2^ii^	0.83 (3)	2.79 (3)	3.513 (2)	147 (2)

Symmetry codes: (i) −*x*, −*y*+1, −*z*+1; (ii) −*x*, −*y*, −*z*.
